# COVID-19 denial in Turkmenistan veiling the real situation

**DOI:** 10.1186/s13690-021-00779-x

**Published:** 2022-01-04

**Authors:** Hashim Talib Hashim, Ahed El Abed El Rassoul, John Bchara, Attaullah Ahmadi, Don Eliseo Lucero-Prisno

**Affiliations:** 1 University of Baghdad, College of Medicine, Baghdad, Iraq; 2 Faculty of Medical Sciences, Lebanese University, Hadath, Beirut, Lebanon; 3 Medical research center, Kateb University, Kabul, Afghanistan; 4 Tishreen University, Faculty of Medicine, Latakia, Syria; 5 Global health Focus Asia, Kabul, Afghanistan; 6 Department of Global Health and Development, London School of Hygiene and Tropical Medicine, London, UK; 7 Faculty of Management and Development Studies, University of the Philippines (Open University), Los Banos, Laguna Philippines

**Keywords:** COVID-19, Humanitarian crisis, Pandemic denial, Turkmenistan

## Abstract

Coronavirus disease 2019 (COVID-19) emerged in late 2019, with the first case identified in Wuhan City, Hubei Province, China, on 12 December 2019. In order to perceive the comprehensive impact of this pandemic, we have to know that misinformation and denials about COVID-19 have surely exacerbated its diffusion and hindered the response against it. Turkmenistan remains one of the very few countries in the world that lacks reports about emerging cases of the novel coronavirus. Turkmen authorities claim that they have adopted all attainable measures required in order to combat the virus, asserting that COVID-19 has yet to reach their country. Despite the government’s reported absence of COVID-19 in the country, rumors, media reports and independent sources suggest the spread of the pandemic in Turkmenistan. By mid-June 2020, the outbreak was referred to as being serious with patients suffering extreme health risks, and following its state of disrepair and unethical practices, many of those anticipated to be COVID-19 infected tend to suffer at home, discouraging any interaction with the healthcare system. The civil society in Turkmenistan, for the time being, takes full part of the government’s duty in the process of informing and educating the public regarding the COVID-19 pandemic, and endeavors to keep the government and WHO accountable for behaving in such repressive ways that could lead to rather preventable loss of human life in Turkmenistan. Yet, efforts hang fire before unveiling the real situation, and Turkmenistan’s government owning up to the negations and roaming speculations, not only regarding the coronavirus crisis, but every public-related issue itself.

## Background

Coronavirus disease 2019 (COVID-19) emerged in late 2019, with the first case identified in Wuhan City, Hubei Province, China, on 12 December 2019. It later expanded exponentially, precipitating an ongoing threat and postulating a global health pandemic affecting day to day life in many countries [1]. In order to perceive the comprehensive impact of this coronavirus pandemic, we have to know that misinformation and denials about COVID-19 have surely exacerbated its diffusion and hindered the response against it, particularly in Turkmenistan. Commencing October 1, 2020, Turkmenistan, with about 6 million citizens, has yet recorded no infections nor any deaths associated with COVID-19. No daily updates are provided and tests are hardly available. However, significant death reports resulting from acute respiratory disorders are found enlisted as consequences of dust and air pollution [2].

Turkmenistan remains one of the very few countries in the world that lacks reports about emerging cases of the novel coronavirus. Turkmen authorities claim that they have adopted all attainable measures required in order to combat the virus, asserting that COVID-19 has yet to reach their country [2, 3]. Despite the government’s reported absence of COVID-19 in the country, rumors are circulating between friends, on social media and within civil society organizations about related cases, while efforts are presumed to be made for monitoring the COVID-related deaths [4]. Various media reports and independent sources also suggest the spread of the pandemic in Turkmenistan (https://www.hrw.org/world-report/2021/country-chapters/turkmenistan). Overall, the government keeps promoting a vagueness affecting the physical and mental health of people in the country, while continuing to apprehend the absence of confirmed COVID-19 cases [4].

This denial of the people’s right for the information, health care and accountability is very common within the borders of Turkmenistan. Adding on, the ongoing denial of an apparent COVID-19 outbreak that further jeopardizes the population’s public health.

### Denial of COVID-19 cases

Turkmenistan is poorly positioned to cope with a pandemic, with public health conditions described as alarming as the country is being ranked 101 out of 195 in the 2019 Global Health Security Index. Turkmen citizens have lost faith in such a decaying healthcare system, and have no belief left in that their government will ever support its citizens’ health [5].

Notably, the government realized the risk of COVID-19 early in the pandemic. In January 2020, flights to countries infected with the virus were suspended and later in February, flights from many infected countries were redirected to Turkmenabat International Airport where passengers were tested for signs of the virus, borders were then closed in March 2020 and those entering Turkmenistan were quarantined. Turkmenistan’s state-run railway declared in July 2020 the suspension of travel by local passenger trains. In August 2020, the suspension was extended, with the authorities announcing the requirement of COVID-free certificates prior to travel once the ban is lifted, such measurements had been implemented as well for air travel. RFE/RL later announced in late 2020 that what essentially amounted to a travel ban had been once again extended to January 2021 [6].

Indeed, the spread of the virus can be ought to the country’s relatively small population and geographic isolation, but the unattainable data and knowledge placed remotely distant from the people aided in having the latter unaware of how to manage themselves and protect their own health, and further conveyed the government’s silence and inability to fulfill its obligations regarding the situation in the country [7, 8].

A WHO expert mission was declared to visit Turkmenistan in April 2020. The 10-day mission took place later in July 2020, as clarified by the organization that the 3-month delay was related to logistical issues. The mission backed up the government’s claims and issued that no confirmed cases are present in the country, but urged the government to behave as if the virus was present, however the WHO’s statement was disappointing and further widens the gap between the Turkmen people and their human rights. Whereupon and as it had been over the years, alternative networks take responsibility to provide the public related health information. SAGLYK (www.Saglyk.org), a non-governmental organization established in 2009, has been providing science-based information on COVID-19 in the Turkmen language to the public since February 2020 [7]. SAGLYK has also issued a public statement on the actions of both; the government and WHO, expressing a great concern that the public health emergency in Turkmenistan has been masked by both sides [9].

The Turkmen government has been known to routinely suppress independent reporting within its borders. Yet several Europe-based organizations with reliable sources inside the country have reported widely about the COVID-19 spread in Turkmenistan. By mid-June 2020, the outbreak was referred to as being serious with patients suffering extreme health risks when an employee with Turkmenistan’s Center for the Prevention and Treatment of Infectious Diseases declared to Radio Azatyk that the fast onset of the deaths of many with correspondent lung damage strongly suggests COVID-19 [10].

SAGLYK had also found out that, until mid-August 2020, Turkmen doctors had obtained no knowledge about the pandemic, medical care or preventative protocols. The lack of COVID-19 testing further eliminates any likelihood of verifying that Turkmenistan is hit by the global pandemic [10, 11].

Rights and Freedoms of Turkmen Citizens, a Prague-based independent group, reported that health workers in Turkmenistan are lacking personal protective equipment (PPE), a shortage that endangers their lives and health. The group further elaborates that it had also received in June reports of Turkmen doctors and nurses being threatened by Turkmen authorities with criminal prosecution and the denial of their right to practice their professions if refusing to serve in facilities with isolated suspected COVID-19 cases. The group had also reported that in July 2020, authorities arrested seven doctors in one region upon their refusal to follow orders and force patients with COVID-19 symptoms to pay for treatment, however by October, the cases against them were dropped and they were released. Turkmen Initiative for Human Rights (TIHR) also reported in the same month a threat by authorities in Turkmenabat to 120 health workers with prosecution after their written plea to the regional administration regarding solving the issue of shortage in PPE and equipment to treat what was described as a pneumonia outbreak there (https://www.hrw.org/world-report/2021/country-chapters/turkmenistan).

What worth is pointing out that Turkmenistan’s claim of being free of the coronavirus is not criticized by many of the countries already hit by the virus, at least not publicly. Guzide Uchkun; the widow of Kemal Uchkun, a Turkish diplomat that passed away in one of Turkmenistan’s hospitals on July 7, 2020, has recently filed a lawsuit against several Turkish government officials upon their failure to transport her husband from Turkmenistan to Turkey to receive proper medical care after the worsening of his symptoms [12], and is planning to file another one against the Turkmen authorities, charging them with negligence and obstruction [13]. Uchkun was admitted to the hospital on June 27, and despite showing signs associated with COVID-19 as Turkish doctors confirmed from the deceased’s x-rays, his wife assures that he was treated for pneumonia and Turkmen doctors were administering him antibiotics that don’t even work against the coronavirus. Moreover, Turkish forensic experts had no doubt that the death was due to COVID-19 and claimed that Uchkun would have survived if he were to be brought back to Turkey, yet Turkish authorities have yet to say anything regarding the diplomat’s death [14].

### Lack of communication

Following its state of disrepair and unethical practices, many of those anticipated to be COVID-19 infected tend to suffer at home, discouraging any interaction with the healthcare system [15]. Testing as well appears to be chaotic with people retaining little information about accessing it. Moreover, the lack of tests and testing equipment provided further limits this access, where those with connections and financial means are able to obtain the test, many who cannot afford it suffer numerous barriers [10, 16].

Many studies on the previous SARS crisis gave much attention to the local organization’s relationship with its public and analyzed the latter’s strategies in implementing communication with respective publics, including employees, media and community [17–19]. Rosenthal and Kouzmin argued in their paper that the roles that the governments play in times of crisis are critical and need to be understood and reflected particularly when the threat, as in a crisis like SARS, exists in the socio-political system [20–22].

The government’s COVID-19 outbreak denial in Turkmenistan however, blocks the route for implementing such investigations, and further wards off expectations to shed light on health crisis communication and the impacts of health communication efforts demonstrated through the different emerging media. The collapse of communication and management has taken the confidence of the public watching neighboring countries’ governments supply their citizens with COVID-19 data, while they receive none to help keep them stay safe at such times of crisis. Efforts to prevent deaths in Turkmenistan are being made by the public and civil society through the WHO and UN agencies assistance in providing humanitarian cargo to medical institutions in the region, yet continue defying human rights and eradicating the people’s trust.

## Conclusion

The civil society in Turkmenistan, for the time being, takes full part of the government’s duty in the process of informing and educating the public regarding the COVID-19 pandemic, and endeavors to keep the government and WHO accountable for behaving in such repressive ways that could lead to rather preventable loss of human life in Turkmenistan.

Practitioners, especially those at governmental levels, can acknowledge the challenges of crisis management, and suggest appropriate strategies to maintain the organization–public communication to resolve the crisis. From this perspective, it is essential to employ a strategy of conflict management in order to examine the history of a health-based social conflict and the actions developed to manage these conflicts. Moreover, it is cardinal to investigate how such social conflicts emerge and settle in a public health crisis, and how various parties involved select strategies to manage the crisis and conflicts. A key strength that allows to understand the dynamic nature of the crisis and to make judgements on what strategies are appropriate at every juncture. Yet, any effort down this path hangs fire before unveiling the real situation, and Turkmenistan’s government owning up to the negations and roaming speculations, not only regarding the coronavirus crisis, but every public-related issue itself.

## Data Availability

Not applicable.

## Abbreviations

WHO: World Health Organization
PPE: Personal protective equipment
TIHR: Turkmen initiative for human rights
